# Supplementary material for: Spatiotemporal filtering method for detecting kinematic waves in a connected environment
Source: PLoS One. 2020 Dec 21;15(12):e0244329. doi: 10.1371/journal.pone.0244329 (PMC7751863; doi:10.1371/journal.pone.0244329)
Supplement: S1 Dataset — (ZIP) [file pone.0244329.s001.zip › Description of sample data set.docx]

**1. Data availability**

The data set to replicate this study are uploaded as Supporting Information.

**2. Description**

This document provides the description of the NGSIM trajectory data-set provided by USDOT
(<https://catalog.data.gov/dataset/next-generation-simulation-ngsim-vehicle-trajectories>, accessed on Aug 25, 2020).
The provided data set are classified by lane, and the data include four lanes from the passing lane (i.e., farthest left lane), which are used in this study. The data is processed to represent the local time and locations of the site (e.g., time 7.8 indicate the 7:48 A.M., June 15, 2005; Local Y indicate the coordinate of the front center of the vehicle with respect to the entry edge of the section in the direction of travel (feet)).

| Column number | Column name | Description |
| --- | --- | --- |
| 1 | Veh_ID | Vehicle identification number (ascending by time of entry into section) |
| 2 | Frame_ID | Frame Identification number (ascending by start time) (1/10 of a second) |
| 3 | Total_frame | Total number of frames in which the vehicle appears in this data set. |
| 4 | Local_time | Time elapsed from the start of the data collection (1/10 of a second) |
| 5 | Time | Time of the day |
| 6 | Local_X | Lateral (X) coordinate of the front center of the vehicle with respect to the left-most edge of the section in the direction of travel. |
| 7 | Local_Y | Longitudinal (Y) coordinate of the front center of the vehicle with respect to the entry edge of the section in the direction of travel. |
| 8 | Veh_Class | Vehicle type: 1 - motorcycle, 2 - auto, 3 - truck |
| 9 | Speed | Instantaneous velocity of vehicle (mile/hour) |
| 10 | Lane | Current lane position of vehicle. Lane 1 is farthest left lane; |
