# Supplementary material for: Spatiotemporal filtering method for detecting kinematic waves in a connected environment
Source: PLoS One. 2020 Dec 21;15(12):e0244329. doi: 10.1371/journal.pone.0244329 (PMC7751863; doi:10.1371/journal.pone.0244329)
Supplement: Attachment — Submitted filename: Response to Reviewers.docx [file pone.0244329.s002.docx]

**PONE_2020_30079 “Spatiotemporal filtering method for detecting kinematic waves in a connected environment”**

**Authors:** Eui-Jin Kim, Koohong Chung*, Dong-Kyu Kim, Seung-Young Kho

The authors really appreciate the anonymous referees who reviewed this paper for their constructive and helpful comments. In this response document, we reponse to each review comments with the label **[Response]**, and corresponding revised section in the manuscript is described with the label **[Manuscript].** We also edited the minor components such as grammar, expressions, and consistency to the revisions.

### Review Comments 1

1. “This paper proposes a spatiotemporal filtering method for detecting kinematic waves in a connected environment. The research topic is interesting and worth of investigation. However, the research gap is not stated clearly in the Introduction or Background Section. The research gap is very important, as it implies the potential contribution of this research.”

**[Response]**

Thank you for your comments. Per the reviewers comments, we have revised background sections to clarify the research gaps in the subsection of the background “Existing techniques for detecting kinematic waves”. A summary of issues from the previous studies and matching them to the improvements of the proposed method has been edited for clarification.

**[Manuscript]**

**pp.6-8**

The above studies focused on investigating spatiotemporal attributes of KWs based on the aggregated behavior of the KWs, rather than detecting individual KW to specify their location and time. Also, their methods commonly require the manual determination of the parameters such as background flow in each road segment [2] and stationary time interval [18].

...

The performance of the above-mentioned studies was evaluated based on visual inspection of the vehicle trajectory data without considering false positives from the random fluctuations. Therefore, the robustness of these methods against random fluctuations in the data had not been evaluated. Such random fluctuations in practice can result in high false positives in detecting KWs.

...

Several issues for detecting KWs can be drawn from the above review of previous studies. First, a data-driven method for detecting KWs needs to be implemented without local calibration of the parameter. This generalizable procedure is an important factor in enabling it to be used in practice. Second, previous studies focus on amplifying the signal of interests, rather than removing random fluctuations that cause false positives in detecting KWs. The impact of random fluctuation would be more prominent in the data from CVT that is potentially contaminated by physical barriers and frequency interference of the data collection system. Last, previous studies conducted only visual inspection for evaluating detecting performance without quantitative measures. Quantitative evaluation is essential for implementing the KW detection method in practice. As a remedy, this paper proposes a data-driven method for detecting KWs. While the methods mentioned above dealt with random fluctuations in the data by amplifying the signal of interest using different window sizes, our proposed method focuses on removing random fluctuations using a sequence of steps. All the parameters in each step can be calibrated only from the data without the subject judgment of the researcher. To evaluate the applicability of the proposed method in practice, we evaluated our method using quantified measures under different CV penetration and data resolution. More details are explained in the later sections.

2. “According to the Data Section, the data on vehicle trajectories are collected on June 15, 2005 from 7:50 to 8:35 A.M. They are too old, and the observation period is short. The empirical analysis conducted on the data observed in recent years and during a long period (such as a week or a month) is preferred.”

**[Response]**

Thank you for your comments. We looked for more recent vehicle trajectory data, however, data at this resolution is rare, and not available. The trajectories of all the vehicles in the road, which we used, are obtained from several video cameras that are synchronized at mounted on top of high buildings adjacent to the roadway. Such data cannot be obtained from unmanned aerial vehicles that have been proposed to provide the images above the traffic since it is difficult to stably provide such 610 m long and 45 minutes of high-resolution video. Especially for the analysis that consider high-resoultion speed and flow, accurate tracking of vehicles in a congested areas is challenging problem due to false dections and tracking errors caused by road environments, adjacent vehicles, and lighting conditions (Kim et al., 2019). To the best of our knowledge, the vehicle trajectory data of NGSIM is the only open-source data that include all trajectories of vehicles on the congested highway and the data of which the quality has been recognized in many microscopic traffic flow studies. We also wanted to verify the proposed emthod in various locations through more long and recent data, but it was unfortunately impossible due to data limitations. If the connected vehicle can provide such data in the future, further verification of this study would be possible. We mentioned those limitations of the data in the “Concluding remarks” section with future research.

**[Manuscript]**

**p.31**

Data availability is also the limitations of this study. Obtaining trajectories of all the vehicles on the road is so difficult since it is extracted from videos of several synchronized cameras mounted on top of high buildings adjacent to the roadway. To the best of our knowledge, the vehicle trajectory data of NGSIM is the only open-source data that include all trajectories of vehicles on the congested highway. Recently, unmanned aerial vehicles have been proposed to provide the images above the traffic, but it is difficult to stably provide such 610 m long and 45 minutes of high-resolution video (Kim et al., 2019). Applying the proposed method to other trajectory data that mimic the data from CVT, or to real CV data would be the necessary and direct subject of future study.

3. “The logistic regression proposed for identifying the KW is somewhat simple. As the observed data are spatially and temporally correlated, accounting for the spatial and temporal correlations in the logistic model is suggested. The following is some representative works on spatial and temporal regression models applied in the field of transportation engineering for the references”
   [1] Investigating the impacts of real-time weather conditions on freeway crash severity: A Bayesian spatial analysis. International Journal of Environmental Research and Public Health, 2020, 17(8), 2768.
   [2] Jointly modeling area-level crash rates by severity: A Bayesian multivariate random-parameters spatio-temporal Tobit regression. Transportmetrica A: Transport Science, 2019, 15(2): 1867-1884.
   Their findings indicate that accommodating spatial and temporal correlations can significantly improve model fit and reduce model misspecification.

**[Response]**

Thank you for your comments on the references. Although the representative works you mentioned cannot be applied directly to our data, we cited thses works as representative works for logistic regression in the transporation field. We have evaluated the performance of using a mixed-effect logistic regression (MELR) model for the recommendation. Utilizing this model allows us to detect the additional number of true kinematic waves and to estimate model covariance among different lanes. The reason why we use this model is descrbied as follows.

The observed data that are used to model the logistic regression is the aggregated flow and speed at the specfic downstream location. The data only include the data-points of valleys in the speed, which are candidate KW as shown in Fig 6. In this setting, sptial dimension is the four lane and the temporal dimensions is 45 minutes of the data. To investigate the spatiotemporal correlation, we present the residual plots of the original logistic regression model as in figure above, prior to developing functional form of mixed effect logistic regression model. The data suggested a stronger presence of spatial correlation (i.e., lane-specific effects) and such that we included the mixed effect model that considers the correlation among the lanes. The temporal correlation is not strong as spatial correlation since the data points (i.e., the valleys that are candidate KWs) are temporally separated at least 2 minutes (i.e., period of the valleys). Therefore, temporal correlation was not included in the model.

The estimation results of MELR model shows significant and reasonble parameters for *H, C,* and random intercepts. The detection performances better than simple logistic regression model were also achieved.

Also, we revised all the results (i.e., detection performance, estimated parameters), findings, and figures (i.e., Figs 7 and 8) concerning the MELR model in the revised manuscripts.

**[Manuscript]**

**pp.21-22**

Logistic regression models have been used by previous studies for analyzing categorical output data [38,39], and advanced models considering characteristics of data such as spatial and temporal correlation also have been proposed [40,41]. This study developed the logistic regression model that includes H and the level of congestion, C, which account for the effects of vehicle spacing related to the generation of waves from instability and lane changes in congested traffic [28]. The value of C was computed as a normalized speed at the valley of candidate KW.

The speed and flow relation across different lanes would be different due to various factors such as the traffic composition and proximity to on-off ramps [36]. To consider the effects of lane-specific characteristics, we include the random effects parameter in the mixed effect logistic regression (MELR) model [42,43]. The proposed MELR models assume that the probability of being KW within lanes may be correlated. The probability that candidate KW is the KW given $\eta$ in the MELR is described as in Eq (9).

$$\eta=\boldsymbol{X\beta}+\boldsymbol{Zu}$$

(9)

$$p\left( Y=1|\eta\right)=\frac{exp(\eta)}{1+exp(\eta)}$$

(10)

where the input data ***X*** is a *N×*3 matrix of the 2 independent variables (i.e., *H* and *C*) and intercept; *N* is the number of data points; $\boldsymbol{\beta}$ is 3×1 vector of the parameters for fixed effects; $\boldsymbol{Z}$ is *N×*4 matrix for 4 different lanes; $\boldsymbol{u}$ is 4×1 vector of the random effects. The components of $\boldsymbol{\beta}$ are respectively estimated parameters for the *C,* *H,* and intercept (i.e., $\beta_{1}$, $\beta_{2}$, and $\beta_{0}$), and ***u*** is distributed as normal with zero mean and variance *V* that is the variance-covariance matrix of the random effects. Since we includes the random effects as deviations from the fixed intercepts effects ($\beta_{0}$), *V* is the variance of random intercept effects. The MELR model is estimated based on the Laplace approximation to the log-likelihood. More details about computational approach for model estimation are presented in [43]. We performed the repeated 5-fold cross-validation 10 times to evaluate the performance of MELR model. The candidate KWs were selected as a KW when the $p\left( Y=1|\eta\right)>0.5$ (Equation 10), and the results of applying the proposed method to the study site are reported in the following section.

4. Besides, more discussions on the results and the practical implications of the findings are expected.

**[Response]**

Thank you for the comments. Per the reviewer's comments, we have included additional examples of practical implications of the findings from our study. We revised the “Concluding remarks” sections to comment on.

**[Manuscript]**

**pp.29-30**

The proposed method obtained a 92.5% F1 score in detecting KW using 5-second aggregated data from a CV in the full connectivity, and it obtained a 91.1% F1 score in partial connectivity. These findings showed strong promise for the proposed method in detecting KW that can be instrumental in developing a real-time control strategy of connected vehicles [24, 25]. Several practical implications can be drawn from the findings of this study. Most of the falsely-identified KWs were emanated near D, while the bottleneck remained active. (See Fig 6(e).) This may be the result of KWs not yet becoming stable since this occurs as they travel further upstream [3,16,37]. Therefore, when the proposed method is applied in practice, the several RSUs collecting traffic data should be installed at specific intervals to detect the downstream’s false negatives in the upstream. Performance evaluation in the various data conditions was used to investigate the impacts of the type of data, temporal aggregation, and penetration rate on detecting KW, and it revealed that high temporal resolution is an important condition of the data for detecting KW. Although data aggregation can reduce the noise of the data (i.e., candidate KWs), the missing attributes of traffic flow is more significant in detecting KWs. Also, the proposed method well-performed 5-second aggregated data even they collected from partial connectivity and loop detector. Therefore, when designing a data collection system for CV, transportation agencies should put a lot of effort into obtaining a high temporal resolution of the data, and the random fluctuation of those data can be effectively removed by denoising methods such as EEMD.

### Review Comments 2

1. How the data of the trajectories of individual vehicles in all of the lanes along the 640-m section of the freeway were collected? The authors could refer to the following one if the video technique is used.
   [1] “Using the visual intervention influence of pavement markings for rutting mitigation-part I: preliminary experiments and field tests”, International Journal of Pavement Engineering, 2019, 20(6), 734-746.

**[Response]**

Thank you for the comments. As you mentioned, the vehicle trajectory data is obtained by video analysis technique. The videos from several synchronized camera record vehicles passing through the study area, and those images are post-processed to extract the vehicle positions on the road every 0.1 seconds. We addered reference about how the trajectories is obtained including your recommending paper.

**[Manuscript]**

**p.8**

The Next-Generation Simulation (NGSIM) project [23] collected data of the trajectories of individual vehicles in all of the lanes along the 640-m section of the freeway shown on the left side of Fig 1(a). The vehicle trajectory data were extracted by automated vehicle tracking system based on video analysis technique [21,24] using videos from several synchronized cameras [25]. The data were collected on June 15, 2005 from 7:50 to 8:35 A.M., and the data included the trajectories of 6,101 vehicles. Four lanes from the far left were used for this study and those data were uploaded as the supporting information in S1 Dataset.

2. More references of logit models are needed. For example, the following ones. [2]  “Investigating the Differences of Single- and Multi-vehicle Accident Probability Using Mixed Logit Model", Journal of Advanced Transportation, 2018, UNSP 2702360.
   [3] Analysis of hourly crash likelihood using unbalanced panel data mixed logit model and real-time driving environmental big data. 2018, JOURNAL OF SAFETY RESEARCH. 65: 153-159.

**[Response]**

Thank you for the comments. Per the reviewer's comments, we have added your recommended references about logit models that applied to other transportation fields.

**[Manuscript]**

**p.21**

Logistic regression models have been used by previous studies for analyzing categorical output data [38,39], and advanced models considering characteristics of data such as spatial and temporal correlation also have been proposed [40,41].

3. The definition of the figures is not high enough.

**[Response]**

Thank you for the comments. We revised the definition of the figures more cleary and specfically as your suggestions.
